# Ouabain potentiates the antimicrobial activity of aminoglycosides against *Staphylococcus aureus*

**DOI:** 10.1186/s12906-019-2532-6

**Published:** 2019-06-06

**Authors:** Neelam Kumari, Snehlata Singh, Vandana Kumari, Siddhartha Kumar, Vinay Kumar, Antresh Kumar

**Affiliations:** 1 Department of Biotechnology, Central University of South Bihar, Panchanpur, Gaya, Bihar 824236 India; 2 National Institute of Plant Genome Research (NIPGR), New Delhi, India

**Keywords:** Ouabain, *Staphylococcus aureus*, Synergism, Aminoglycosides, Gentamycin uptake, Biofilm inhibition, Antimicrobial activity, Biofilm detachment

## Abstract

**Background:**

*Staphylococcus aureus* is a notorious pathogen which often causes nosocomial and community attained infections. These infections steadily increased after evolving the resistance due to indecorous practice of antibiotics and now become a serious health issue. Ouabain is a Na^+^/K^+^-ATPase inhibitor that leads to increase the heart contraction in patients with congestive heart failure.

**Methods:**

In the present study, in vitro antimicrobial effect of ouabain together with aminoglycosides was determined against clinical and non-clinical *S. aureus* strains*.* Using checkerboard, Gentamycin uptake and biofilm assays, we analysed he interactions of ouabain with aminoglycosides.

**Results:**

Ouabain induced the staphylocidal potency of aminoglycosides by remarkably reducing the MIC of gentamycin (GEN) by 16 (0.25 μg/mL), 8 folds (0.5 μg/mL) amikacin (AMK); and 16 folds (1.0 μg/mL) with kanamycin (KAN), compared to their individual doses. OBN severely reduced cell viability within 60 min with GEN (1 μg/mL), KAN (2 μg/mL) and 90 min with AMK (1 μg/mL). This bactericidal effect was enhanced due to GEN uptake potentiated by 66% which led to increase the cell permeability as revealed by leakage of bacterial ATP and nitrocefin assay. The biofilm adherence disrupted by 80 and 50% at 5 mg/mL and 1.5 mg/mL OBN and 50 and 90% biofilm formation was inhibited at 5 mg/mL (MBIC_50_) and 10 mg/mL (MBIC_90_), respectively. Moreover, OBN with GEN further induced biofilm inhibition by 67 ± 5% at pH 7.0.

**Conclusions:**

Taken together, we established that OBN synergizes the antimicrobial activity of aminoglycosides that induces cell killing due to intracellular accumulation of GEN by disturbing cell homeostasis. It may be proven an effective approach for the treatment of staphylococcal infections.

## Background

Superficial and invasive infections caused by *Staphylococcus aureus* continue to raise serious health challenges globally. These acute and chronic infections have now become more problematic after emerging multi-drug resistance (MDR) against various frontline antibiotics [1]. The problem of MDR in *S. aureus* is subsequently emerging both in nosocomial and hospital-acquired settings, with a significantly higher mortality and morbidity rate [2, 3]. The condition of drug resistance is primarily developed by unregulated sales of antibiotics, a long course of medication, indiscriminate usage of drugs and poor public health infrastructure. Statistically, incidences of methicillin-sensitive *S. aureus* (MSSA), methicillin-resistant *S. aureus* (MRSA) and vancomycin-resistant *S. aureus* (VRSA) infections have been endemic as it steadily increased up to 54% [4–6]. According to Indian hospitals survey, over 80% clinical samples of *S. aureus* were established resistance to the frontline antibiotics including methicillin [7]. However, the prevalence of MSRA infection in the U.S. accounts for 94,000 cases and over ~ 18,000 deaths per year [8]. Despite it, readily biofilm formation on the medical devices and host tissues also contribute to the persistent chronic infections. Biofilm embedded *S. aureus* remarkably decreased antibiotic and immune-defence susceptibility by over 100 folds and making them difficult to treat clinically [9, 10]. The process of biofilm formation is multifactorial among which polysaccharide intercellular antigen (PIA) synthesized from UDP-N-acetylglucosamine via intercellular adhesion (*icaADBC*) locus play an important role. It has been reported that point mutant in the *icaADBC* locus abrogated the capacity of biofilm formation in *S. aureus* [11]. In contrast, secretions of virulence factors during different growth phase also contribute to biofilm formation [12]. Combating this notorious infection remains a major challenge as most of the conventional antibiotics have now become redundant to work and an immediate treatment regimen required for its elimination. Much efforts have gone into devising a workable treatment against staphylococcal infections particularly for the elimination of MRSA, VRSA pathogens by 1) searching a new antimicrobial from different sources 2) repurposing a new therapeutic property of the known drugs 3) synergizing the efficacy of the antibiotics with combination of others. Identification of the new antimicrobials via de novo synthesis and screening are a slow, costly and traditional approach. Most of the on-going research work to identify a new antimicrobial is focused on to repurposing existing drugs with known therapeutic property and toxicity that remarkably reduce treatment cost and side-effects with an antibiotic development. Simvastatin which originally used for the treatment of cardiovascular disease as it decreases the cholesterol level has also showed antimicrobial property against list of Gram-positive pathogens [13]. Similarly, anti-inflammatory, anti-oxidant ebsleen, antineoplastic 5-fluoro-2′-deoxyuridine (FdUrd) and anti-rheumatoid auranofin have also been reported to possess a strong bactericidal effect on drug-resistant; MRSA and VRSA strains [13, 14]. Combining two or more therapeutic agents is another lucrative approach for synergizing treatment and promptly elimination of pathogens by reducing the antibiotics load of the individual drug. Interactions between different antibiotics pair were found be synergistic against MSSA, MRSA, and Pseudomonas acquired infections. Plectasin paired either with β-lactam or aminoglycoside**,** Vancomycin increased potency of gentamycin, Nordihydroguaiaretic acid enhanced antimicrobial activity of aminoglycosides, glycerol monolaurate **(**GML) and lauric acid either with streptomycin or gentamicin are such examples [15–17]. In addition, the synergistic interaction of antibiotic with a variety of different compounds has also been reported. For example, hydroisothiocynates synergistically inhibited the growth of *S. aureus* with streptomycin. The polymyxin B boosted doxycycline and trgecycline activity against doxycycline-resistant and susceptible *K. pneumoniae* clinical isolates [18].

Ouabain or g-strophanthin is a cardiotonic steroid [19], derived from the plants (*Strophanthus gratus, Acokanthera schimperi*) or secreted endogenously by the adrenal glands [20]. It acts on α-subunit of Na^+^/K^+^-ATPase to inhibit its transport activity resulting in an increase intracellular sodium ion (Na^+^) concentration [21, 22]. The physio-pathological role of ouabain is linked to increase the heart contraction in patients with congestive heart failure [23]. However, disruption of Na^+^K^+^-ATPase activity leads to disturb membrane polarity with subsequent accumulation of intracellular Ca^2+^ level and neurotransmitter release [24]. A group of studies has demonstrated that ouabain also promotes different types of cell proliferation [25–29] and cell susceptibility against different microbes [30]. However, the efficacy of ouabain on persistent *S. aureus* biofilm and functional mechanism for the treatment of Staphylococcal infection has not elucidated so far. With this background, the aim of the present study is to evaluate in vitro activity of ouabain together with aminoglycosides (GEN, KAN, AMK) and other antibiotics (AMP, TET, VAN) against *S. aureus.*

## Methods

### Chemicals, Bacteria strains, and media

Ouabain (OBN) and ATP (adenosine 5′-triphosphate), FITC (fluorescein isothiocyanate), Crystal violet, used in this study were purchased from the Sigma Aldrich (St. Louis, MO). Antibiotics such as ampicillin (AMP), tetracycline (TET), vancomycin (VAN), gentamycin (GEN), amikacin (AMK) and kanamycin (KAN) was procured from HiMedia (Mumbai, India).

The methicillin-sensitive *S. aureus* (MSSA); ATCC29213 was used in this study. Clinical *S. aureus* strains IGMS 002 and IGMS 007 received a kind gift from the Indira Gandhi Institute of Medical Science (IGIMS), Patna, were used for cell susceptibility analysis. Muller Hinton Agar/Broth (MHA/MHB) and Tryptic Soya Broth (TSB) were used to culture *S. aureus* and biofilm formation, respectively at 37 °C. Culture media was purchased from the Hi-media Laboratory.

### Spot assay

The antimicrobial susceptibility of OBN alone or in combination of tested antibiotics (AMP, TET, VAN, GEN, AMK and KAN) was determined as described earlier [31, 32]. Briefly, both ATCC 29213 and clinical isolates (IGMS02 and IGMS07) of *S. aureus* were cultured in Muller–Hinton broth (MHB) till the cell density achieved the exponential stage (OD_600nm_ reaches 0.5). Cells were then resuspended into 1X phosphate buffer saline pH 7.4 (PBS) to maintain cell density 0.1 OD_600nm_ (2 × 10^8^ cells/mL) for spotting. 5 μL of five folds serial dilutions were spotted on MHA plate containing AMP (0.125 μg/mL), TET (1.5 μg/mL), VAN (0.5 μg/mL), GEN (1.0 μg/mL), AMK (1.0 μg/mL), KAN (1.0 μg/mL) alone or with 1 mg/mL OBN. The difference in *S. aureus* cell growth was observed after incubation for 16 h at 37 °C.

### Chequerboard assay

The interaction of OBN with panel of antibiotics; AMP, TET, GEN, AMK, GEN, and KAN, was determined by the chequerboard method as described elsewhere with minor modifications [33]. The interactions between two tested antibiotics combinations were represented by fractional inhibitory concentration (FIC) index for each agent. The FIC can be expressed as the sum of MIC (minimal inhibitory concentration) of two tested agents in combination divided by the MIC of the individual agent. Briefly, two folds of serial dilutions of tested antibiotics were made (mg/mL) such as 0.062–32 (GEN and AMK), 0.00025–0.128 (KAN), 0.000004–0.002 (AMP), 0.000016–0.008 (VAN and TET) and 1.4–128 (OBN) and latter 100 μL (5 × 10^4^ cells/mL) *S. aureus* cells suspension was added to each well and cultured for 48 h at 37 °C. The MIC value of each tested agent was observed by measuring the optical density at OD_600nm_. Each chequerboard test generates many different combinations and by convention, the FIC value of the most effective combination is used by calculating the fractional inhibitory concentration index (FICI). FICI was calculated by adding both FICs:

$$ \mathrm{FICI}={\mathrm{FIC}}_{\mathrm{A}}+{\mathrm{FIC}}_{\mathrm{B}}=\left({{\mathrm{C}}_{\mathrm{A}}}^{\mathrm{comb}}/{{\mathrm{MIC}}_{\mathrm{A}}}^{\mathrm{alone}}\right)+\left({{\mathrm{C}}_{\mathrm{b}}}^{\mathrm{comb}}/{{\mathrm{MIC}}_{\mathrm{B}}}^{\mathrm{alone}}\right) $$

Where, MIC A alone and MIC B alone are the MICs of drugs A and B when acting alone and C_A_^comb^ and C_b_^comb^ are concentrations of drugs A and B at the isoeffective combinations, respectively. Synergism interactions between two tested agents were interpreted in terms of FICI value when it is < 0.5, indifferent between ≥0.5 to < 2, and antagonistic when it was 4 [34, 35].

### Cell viability assay

To analyse the staphylocidal activity of Ouabain (OBN) in combination with tested antibiotics, 2 × 10^6^ mid-log phase cells were taken as described elsewhere [31]. The cells were treated with a fix 1 mg/mL OBN, in absence or presence of 0.25X MIC of GEN (1.0 μg/mL), AMK (1.0 μg/mL) and KAN (2.0 μg/mL). After the cell treatment, it was kept at 37 °C at 120 rpm. 10 μL of cell fractions were taken intermittently after each 2 h intervals. Cell fractions were serially diluted up to 1000 folds with 1X PBS to reduce drug concentration. The viable colonies were calculated after overnight incubation at 37 °C. Percentage of cell killing of treated samples was calculated with respect to the untreated samples (control) using formula (No. of CFU obtained in treated sample/ No. of CFU obtained in control sample) × 100.

### Membrane permeability assay

The membrane permeabilizing property of OBN against *S. aureus* was determined by two different methods mentioned hereunder: **ATP leakage assay:** Briefly, *S. aureus* cells were harvested to mid-exponential phase and diluted to maintain 1 × 10^6^ cells/mL in 50 mM Tris-Cl, pH 7.5; 135 mM NaCl supplemented with 1 mg/mL OBN in absence or presence of 1 μg/mL GEN. Cells were also treated with 35 μg/mL Gramicidin B, which acts as a positive control. During analysis, 100 μL sample was withdrawn at 15 min intervals and supernatants were recovered by centrifugation for measurement of ATP leaks out during treatment [16]. The amount of ATP leakage was quantified upon its hydrolysis into inorganic phosphate at 750_nm_. The ATPase assay was perfumed using purified Nucleotide Binding Domain 1(NBD-1) of Candida drug resistance 1(Cdr1) protein of *Candida albicans* [36]. **Nitrocefin disc assay:** The cell permeabilization of *S. aureus* was determined by measuring the nitrocefin hydrolysis assay by cellular beta-lactamase as described elsewhere [37]. Briefly, overnight aged *S. aureus* cells were treated with 1 mg/mL OBN alone or in combination with 2 μg/mL GEN. During analysis, 100 μL sample was withdrawn after 60 min and supernatant was recovered by centrifugation at 10,000 rpm. Alteration in colour from yellow to red shows nitrocefin hydrolysis which was monitored spectrophotometrically at 485_nm_ and also quantified yellow to red disc colour change using Image J.

### Gentamicin (GEN) uptake assay

The cell culture was harvested to mid-exponential phase and cell suspension was made to maintain cell concentration 0.5 OD (1 × 10^8^ cells/mL) with 1X PBS. The cells were kept on starvation at 37 °C for 1 h on mild shaking conditions which were treated with 2 mg/mL OBN for 45 min at 37 °C in the absence or presence of 10 mM Glucose followed by addition 1 μg/mL GEN alone or in combination with crude cell membrane isolated from *S aureus.* Cells were treated further for 15 min. The supernatant was recovered by centrifugation (8000 rpm, 2 min at 4 °C) after 5 min incubation on ice. Both starved and non-starved cells were used for GEN uptake. Absorbance was measured at 202_nm_ to estimate the extracellular GEN concentration [38].

### Preparation of crude cell membrane (CE)

Briefly, 5 mL *S. aureus* cells were harvested to mid-exponential phase and resuspended in 1 mL, 1X PBS containing 1% TritonX-100. Cells were disrupted by sonication at 30 s pulse followed by 1 min rest, repeated 4 cycles on ice. The supernatant was recovered by centrifugation at 4000 rpm and 4 °C for 10 min followed by high-speed centrifugation at 18000 rpm and 4 °C for 45 min [39]. The pellet was suspended in 1X PBS and stored at − 20 °C for GEN uptake analysis.

### Cell attachment assay

The cell adherence effect of OBN was determined by crystal violet staining and FITC labelling. **Crystal violet cell adherence assay:** Briefly, *S. aureus* cells were harvested to mid-exponential phase and diluted to maintain 2 × 10^5^ cells/mL in TSB containing 5 mM glucose. 200 μL cell suspension was taken in sterile 96 wells polystyrene plate. The biofilm attachment was also analysed on sterile glass slide (10 × 10 mm) put in the wells and varying OBN concentration (mg/mL) from 0.5 to 5 were added for 3 h at 37 °C. The OBN untreated *S. aureus* cells were considered as a control. Unbound cells washed with PBS and fixed using 100% methanol for 15 min. The glass slide and plate wells both were stained with 200 μL of 0.1% (v/v) crystal violet for 5 min. Excess stain was gently rinsed off. The biofilm attachment on glass slide was observed under inverted microscope. The optical density of wells was measured at OD_595nm_ in 96 well plate reader after re-solubilization biofilm in 200 μL, 95% (v/v) ethanol [40].

### FITC cell adherence assay

Briefly, 5 mL *S. aureus* cells were harvested to mid-exponential phase to maintain 1 × 10^8^ cells in 1 mL, 1X PBS. For FITC labelling, cells were incubated overnight at 4 °C with FITC (1.0 mg/mL) with gentle stirring and rinsed off unbound FITC with PBS. The glass slide pre-treated overnight at 4 °C in the presence or absence of blood plasma were put into 200 μL, FITC labelled cells seeded in the 24 wells polystyrene plate. The plate was incubated for 30 min at 37 °C with or without OBN (5.0 mg/mL). Thereafter, cover slides were repeatedly washed with PBS and observed under inverted fluorescence microscope [40].

### Inhibition of biofilm formation

The effect of OBN on biofilm inhibition was determined as described elsewhere [31, 41, 42]. Briefly, 2 × 10^5^ cells from the overnight *S. aureus* culture were incubated in glucose (5 mM) containing TSB of three different pH i.e. 7.0, 6.0 and 5.0 for biofilm formation. Biofilm was grown on microliter plate well and sterile glass-slide (10 × 10 mm) placed in 24 wells polystyrene plate. Biofilm was treated with subsequent increasing concentration of OBN ranges from 1 to 5 mg/mL, added individually or with GEN (1 μg/mL) at 37 °C for different time periods (24 h & 72 h). After incubation, both wells and glass slides were washed with 1X PBS to remove planktonic bacteria. The cells were then fixed with 200 μL methanol for 15 min and plates were allowed to dry. The slide and plate well, both were stained with 200 μL of 0.1% (w/v) crystal violet for 5 min. Excess stain was gently rinsed off and plates were air-dried. After staining, biofilm was observed under the inverted microscope or re-solubilization in 200 μL, 95% (v/v) ethanol and concentration was measured at OD_595nm_ for biofilm inhibition analysis. The OBN untreated *S. aureus* cells were considered as a positive control in both experiments.

### Statistical analyses

Statistical analyses were assessed using GraphPad prism 6.0 (Graph Pad Software, La Jolla, CA). *P* values were calculated via Student t-test and Avova test.

## Results

### Ouabain synergizes *S. aureus* susceptibility to aminoglycosides

**Fig. 1:**
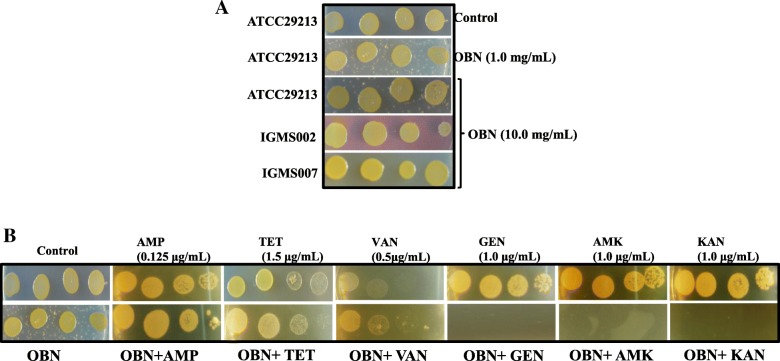
Susceptibility profile of OBN with topical antibiotics. The cell suspension of *S. aureus* were sequentially diluted by five folds and spotted on MHA plate. **a** *S. aureus* ATCC 29213 and clinical strains; IGMS 002 and IGMS 007 spotted in the absence (control) and presence of 1 and 10 mg/mL OBN. **b** Combinatorial effect of OBN in the absence (control) or presence of 0.125, 1.5, 0.5 μg/mL of ampicillin (AMP), tetracycline (TET), vancomycin (VAN), and 1.0 μg/mL of gentamycin (GEN), amikacin (AMK) and kanamycin (KAN) respectively, with 1.0 mg/mL OBN on spotted *S. aureus* ATCC 29213

Ouabain (OBN) is a known Na^+^-K^+^-ATPase pump inhibitor which leads to disturb membrane integrity of the eukaryotic cell. As a result, it causes cell damage via plasma membrane depolarization (PMD). To confirm the killing effect of OBN, spot assay was performed using two different OBN concentrations; 1.0 and 10 mg/mL against methicillin-sensitive *S. aureus* (MSSA); ATCC 29213 and *S. aureus* clinical isolates, IGMS 002 and IGMS 007. No effect on cell susceptibility was observed even though at higher OBN concentration (Fig. 1a). Combining more than one antibiotic is considered to be more effective to control notorious pathogens. To find out whether OBN combined with conventional antibiotics may lead any alteration in drug susceptibility. The activity of OBN in combination with ampicillin (AMP), tetracycline (TET), vancomycin (VAN), and known aminoglycosides; gentamycin (GEN), amikacin (AMK), kanamycin (KAN) were determined using broth microdilution checkerboard assay against *S. aureus*. The fractional inhibitory concentration (FIC) index for these combinations was calculated which was shown in Table 1. In combination with OBN, MIC was remarkably reduced by 16 (0.25 μg/mL), 8 (0.5 μg/mL) 16 folds (1.0 μg/mL) for GEN, AMK and KAN, respectively and thereby demonstrated synergistic effect with aminoglycosides. The 1.0 mg/mL of the lowest concentration of OBN was enabled to stimulate the most synergistic effect with aminoglycosides. Of note, no such synergistic interactions were observed with AMP, TET and VAN. To validate the synergistic effect of OBN with aminoglycosides, spot assay further visualized the interaction of OBN with GEN, AMK, and KAN when a fix 1.0 μg/mL of GEN, AMK, and KAN supplemented with 1.0 mg/mL OBN that It noticeably hyper-susceptible to these aminoglycosides than the individual effects of antibiotics (Fig. 1b). We did not find antagonistic interactions between OBN and any of the drugs tested.

**Table 1:** Checkerboard assay of GEN, AMK, KAN, AMP, VAN, TET and OBN against *S. aureus*

Antimicrobial agents	MIC of each agent (μg/mL)				
	alone	Comb	FIC	FICI	Outcome
GEN	4	0.25	0.0625		
OBN	32000	1000	0.03125	0.0937	Syn
AMK	4	0.5	0.125		
OBN	32000	1000	0.03125	0.1562	Syn
KAN	16	1	0.0625		
OBN	32000	2000	0.0625	0.125	Syn
AMP	0.5	0.25	0.5		
OBN	32000	8000	0.25	0.75	NI
VAN	1	0.25	0.25		
OBN	32000	16000	0.5	0.75	NI
TET	2	0.5	0.25		
OBN	32000	8000	0.25	0.5	NI

*Syn* Synergism, *NI* No Interaction

### Cell viability confirms synergism

**Fig. 2:**
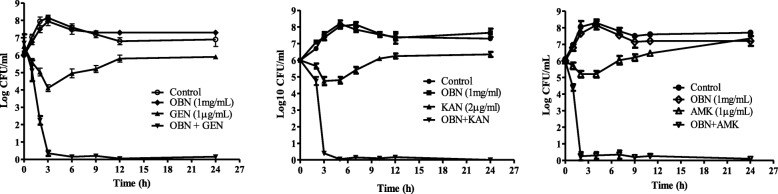
Cell killing effect of OBN with aminoglycosides. The bactericidal effect was measured by treating *S. aureus* ATCC29213 cells for different time course in the absence or presence of 1 mg/mL OBN alone or with GEN (1.0 μg/mL), AMK (1.0 μg/mL) and KAN (2.0 μg/mL). The cell viability was measured by diluting 10uL sample fraction by 1000 folds

To assess the growth inhibition of *S. aureus* was a result of cell killing when OBN supplemented with aminoglycosides (GEN, AMK, and KAN) in the medium. A mid-exponential stage cells were treated with a fix 1.0 mg/mL OBN and 0.25X MIC of aminoglycosides alone or in combination with them. Figure 2 illustrates that GEN (1 μg/mL), AMK (1 μg/mL) and KAN (2 μg/mL) at 0.25X MIC initially affects cell viability which tends to recover after 4 h of the treatment. In contrast, cells were found to be completely killed with no significant viable cells (±2%) within 90 min exposure of OBN, combined either with GEN or KAN respectively and similar effect was also observed within 60 min in presence of OBN supplemented with AMK. No such cell viability was recovered even OBN long exposure (24 h) supplemented with these aminoglycosides. These results ensure that the OBN potentiates bactericidal effect of tested aminoglycosides antibiotics i.e. GEN, AMK and KAN. Of note, the regrowth observed with single used of GEN, AMK and KAN was due to cell adaptation and /or overcome drug stress in the initial stage of growth condition.

### OBN potentiates gentamycin (GEN) uptake

**Fig. 3:**
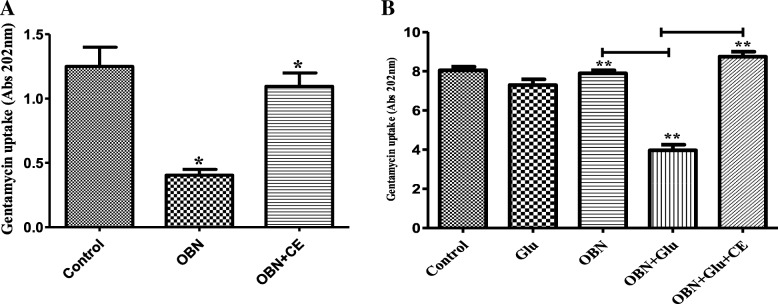
OBN potentiates the gentamycin (GEN) uptake. The GEN (1 μg/mL) treated *S. aureus* ATCC29213 cells were incubated in the absence (control) or presence of OBN (2 mg/mL) and measured extracellular GEN concentration. **a** Mid-log phase active cells used in the absence or presence of crude membrane (CE) with OBN. Two-tailed Student *t* test was performed for statistical analysis. *P* values of (*P ≤ 0.05) are considered as significant. *P* values of control vs OBN: 0.032, OBN vs OBN + CE: 0.026. **b** Starved cells re-energized with 10 mM glucose (Glu) used in the presence and absence of crude membrane (CE). Statistical analysis was performed via the two-tailed Student *t* test. *P* values of (**P ≤ 0.01) are considered as significant. Detailed “P” values are control vs OBN + Glu: 0.0073, OBN vs OBN + Glu: 0.0072, OBN + Glu vs OBN + Glu + CE: 0.0065

It has been well documented that uptake of aminoglycoside the cell is the proton motive force (PMF) dependent. Disturbance in PMF system by inhibiting electron transport severally abolished GEN uptake in eukaryotes. To explore the inhibitory effect of OBN on PMF, GEN uptake was determined using mid-log cells tested under two conditions; non-starved active or starved cells re-energized with glucose. An active mid-log phase *S. aureus* cells were pre-incubated with 1.0 mg/mL OBN for 30 min followed by 10 min GEN treatment. OBN that induced the cell killing with aminoglycosides (GEN, KAN and AMK), also significantly induces GEN uptake by 66% (Fig. 3a) as compared to control (only GEN treated) (*P ≤ 0.05). To further confirm energy dependent GEN uptake, cells were starved for an hour in 1X PBS. The starved cells reenergized with glucose (10 mM) remarkably increased GEN uptake by 50% with OBN, as compared to control. Of note, GEN uptake was found to be limited when starved cells (de-energized) were individually treated with OBN and glucose (Fig. 3b). Statistical analysis was performed via the two-tailed Student *t* test. *P* values of (**P ≤ 0.01) are considered as significant. However similar restriction in GEN uptake was also observed when crude membrane (extracted from *S. aureus*) added into the both tested conditions (Fig. 3a, b). The intracellular accumulation of GEN is responsible for cell killing.

### Effect of OBN on cell permeabilization

**Fig. 4:**
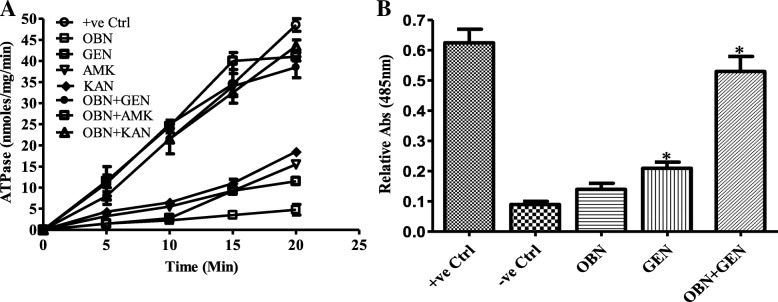
Cell permeability effect of OBN with aminoglycosides. **a** *S. aureus* ATCC29213 cells treated in the absence (−ve control) and presence of OBN (1 mg/mL) and 0.25X MIC of aminoglycosides (GEN, TET, AMK) either added alone or in combination. Gramicidin B was used as a positive (+ve) control. **a** Measured extracellular leakage of cellular ATP by ATPase assay at different time intervals. **b** Nitrocefin hydrolysis was measured with and without (−ve control) addition of GEN with OBN alone or combination after 60 min treatment. Statistical analysis confirmed a significant difference with “P” value, between control vs GEN: 0.033, control vs OBN + GEN: 0.013 and OBN vs OBN + GEN: 0.018

The complex composition of protein and lipids of the cell membrane makes it one of the most significant barriers that selectively allow molecules to pass through it. Any alteration in the cell membrane affects cell permeability. To confirm whether increased GEN uptake is the result of membrane alteration, cell permeabilization effect was determined by measuring the leakage of cellular ATP and hydrolysis of nitrocefin by whole cell. The membrane permeabilizing property of the OBN against *S. aureus* cell membrane, ATP leakage was measured using purified NBD1 ATPase protein. When cell membrane becomes more permeable, more amounts of ATP releases outside the cell that can be corroborated with ATP dependent-ATPase activity. During analysis, different samples were withdrawn at 5 min intervals and supernatants used for measurement of leakage of cellular ATP. Cells treated with 0.25X MIC aminoglycosides (GEN, KAN and AMK) and OBN alone didn’t significantly alter the cell permeability as low ATP activity was observed even at 20 min cells exposure. Similar to the positive control, 0.25X MIC of tested aminoglycosides; GEN (1.0 μg/mL), AMK (1.0 μg/mL), KAN (2.0 μg/mL) along with 1 mg/mL OBN treated cells exhibited a higher time-dependent activity with 35 ± 5 nmoles/mg/min ATP activity at 20 min of exposure (Fig. 4a). The similar effect of OBN with GEN was also verified by nitrocefin assay as shown in Fig. 4b. These results indicate that leakage of more ATP is due to increased cell permeability. *P* values of (*P ≤ 0.05) are considered as significant difference via the two-tailed Student *t* test.

### OBN clears up the bacterial cell adherence

**Fig. 5:**
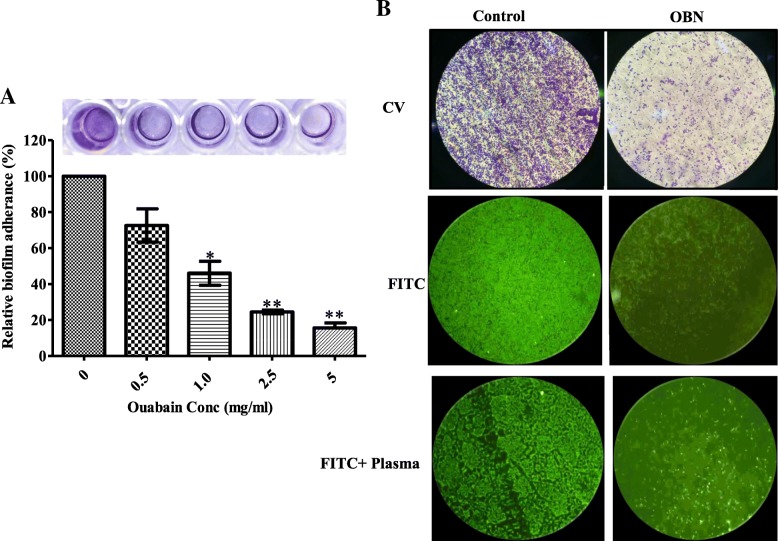
Biofilm cell detachment effect of OBN. *S. aureus* ATCC29213 cells treated without (control) or with substantial increasing OBN concentration ranges from 0.5 to 5 mg/mL for 3 h. **a** The cell detachment was measured by spectrophometrically at 595_nm_. The results are given as means± SD (*n* = 3). *P* values of (*P ≤ 0.05) (**P ≤ 0.01) are considered as significant in comparison to control groups. Detailed “P” values are 0 vs 1.0: 0.015, 0 vs 2.5: 0.0002, 0 vs 5.0: 0.0011

Formation of biofilm over the cell boundary and expression of cell wall associated factors are accountable for pathogens adherence on different host tissue. Such bacterial adherence promotes the cell infections and causes a major hurdle in the antimicrobial treatment. To investigate the OBN effect on disruption of cell adherence (detachment), microliter dish biofilm assay was performed. Figure 5a illustrates that the biofilm attachment to the solid surface (polystyrene plate) was lost almost 80 and 50% when early exponential cells were treated with 5 mg/mL and 1.5 mg/mL OBN, respectively. The loss in cell attachment was further corroborated with FITC labelled and or blood plasma coated *S. aureus* cells (Fig. 5b). As expected, OBN remarkably lost the cell adherence ability of *S. aureus* on the solid surface. Statistical analysis was performed via the two-tailed Student *t* test. *P* values of (*P ≤ 0.05, & **P ≤ 0.01) are considered as significant difference. These studies clearly demonstrate the role of OBN in the disruption of cell attachment to the adhered surface.

### OBN inhibits the biofilm formation

**Fig. 6:**
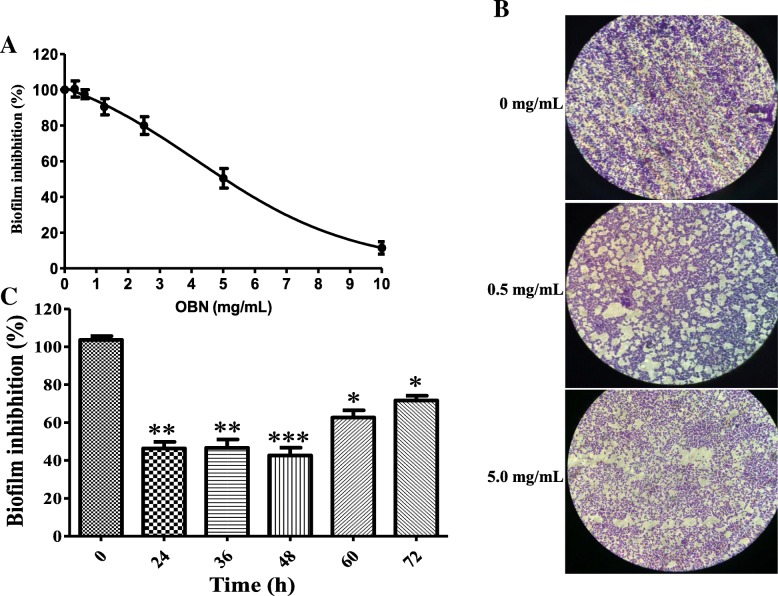
Determination of biofilm inhibitory effect of OBN: **a** Matured biofilm of *S. aureus* ATCC29213 strain was treated with treated with increasing OBN concentration (1–10 mg/mL) and minimal biofilm inhibitory concentration 50% (MBIC_50_) and 90% (MBIC_90_) was measured at 5 and 10 mg/mL, respectively. **b** Time-dependent biofilm inhibitory effect of OBN. **c** Microscopic analysis of inhibition of biofilm formation at 0.5 and 5 mg/mL OBN. Statistical analyses were done using the two-tailed Student t test. *P* values of (*P ≤ 0.05) (**P ≤ 0.01) (***P ≤ 0.001) are considered as significant in comparison to control in groups. Detailed “P” values are 0 vs 24: 0.007, 0 vs 36: 0.005, 0 vs 48: 0.001, 0 vs 60: 0.0179, 0 vs 72: 0.02

To further evaluate OBN effect associated with the inhibition of biofilm formation, *S. aureus* cells grown in biofilm forming TSB medium with varying OBN concentration and biofilm mass was measured after 2 h of treatment. Figure 6a illustrates that the biofilm formation was inhibited by 50 and 90% at 5 mg/mL (MBIC_50_) and 10 mg/mL (MBIC_90_) OBN, respectively after 24 h of OBN treatment. The biofilm inhibitory effect of OBN was further confirmed by microscopic analysis. Cells treated with 0.5 mg/mL and 5 mg/mL OBN reduced the biofilm cell aggregated by approximately 10 and 50%, respectively compared to untreated (control) (Fig. 6b). Of note, the inhibition of biofilm formation tends to increase upon longer exposure greater than 48 h of OBN treatment (Fig. 6c). *P* values of (*P ≤ 0.05), (**P ≤ 0.01) and (***P ≤ 0.001) are considered as significant difference using two-tailed Student *t* test. These results indicate that OBN inhibits biofilm formation at the initial stage more efficiently than the preformed matured one.

### pH-dependent biofilm inhibition effect of OBN

**Fig. 7:**
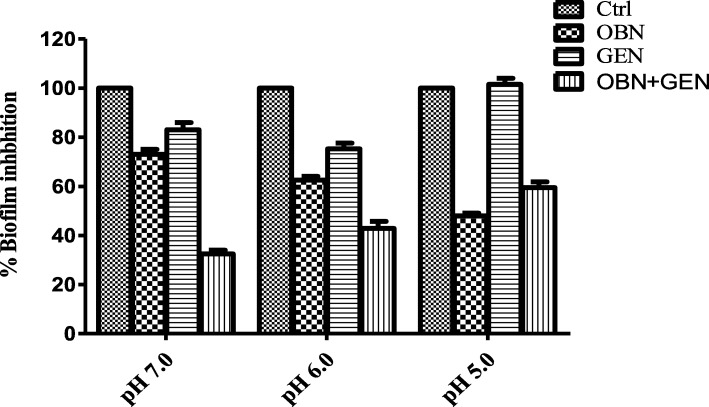
pH dependent biofilm inhibition effect of OBN. The pH-dependent inhibition of biofilm formation was determined by treating *S. aureus* ATCC29213 cells in the absence (control) and presence of 1 mg/mL OBN and GEN (1.0 μg/mL) either added alone or with their combinations at three 5.0, 6.0 and 7.0 pH. Statistical analyses were done using the two way Anova test. *P* values of (*P* ≤ 0.05) (*P* ≤ 0.01) are considered as significant in comparison to control groups. Detailed “P” values are Ctrl vs OBN 0.003, Ctrl vs Glu: 0.02, Ctrl vs OBN + Glu: 0.01

Henry-Stanley et al., investigated that the biofilm formation steadily decreases under acidic pH. The pH-dependent biofilm formation effect was investigated in the presence of OBN at three pH conditions i.e. pH 7.0, 6.0, 5.5 which was normalized by cell viability. Biofilm grown for 24 h was treated with 1 mg/mL OBN and 1 μg/mL GEN individually or together. Biofilm formed was firmly adhered to the polystyrene coated plate at pH 7.0 and it was loosely packed at acidic pH. It was substantially reduced by 21 ± 4%, 37 ± 5% and 55 ± 5% at 7.0, 6.0 5.5 pH respectively when treated with OBN. At pH 7.0, the biofilm was synergistically reduced by 67 ± 5% and no similar effect was observed at pH 6.0 and 5.5 when both OBN combined with GEN. Of note, *S. aureus* exhibited resistance to GEN at lower pH (Fig. 7). Statistical analyses were done using the two way Anova test. *P* values of (*P* ≤ 0.05) (*P* ≤ 0.01) are considered as significant.

## Discussion

The therapeutic utilities of OBN have been explored against different medical issues [24, 25, 28] but its biofilm inhibitory activity and antimicrobial synergism with topical antibiotics have unrevealed. In present in vitro study, we found that OBN boosted up the antibacterial activity of aminoglycosides and also inhibits biofilm formation. To the best of our knowledge, it is the first time showing synergy between OBN and aminoglycosides against clinical and non-clinical *S. aureus* strains. No significant antimicrobial activity of OBN alone was observed against *S. aureus* with a MIC 32 mg/mL. When OBN combined with aminoglycosides such as GEN, AMK, KAN, synergistic interactions were observed which was confirmed by microdilution checkerboard assay. These combinations remarkably reduced the MIC by 16 (0.25 μg/mL), 8 (0.5 μg/mL) 16 folds (1.0 μg/mL) for GEN, AMK and KAN, respectively. In contrast, a FICI value > 0.5 showed no similar interactions with AMP, VAN, TET to OBN. The synergistic boot up of OBN with aminoglycosides was further confirmed by spot analysis and also corroborated by cell viability analysis over time. The cells were completely eliminated with no significant viable cells (±2%) within 90 min OBN (1 mg/mL) treatment, combined either with GEN (1 μg/mL) or KAN (2 μg/mL) respectively and 60 min in the presence of AMK (1 μg/mL). Remarkably, the cell growth remained to suppress with no viable cell recovery observed even after 24 h long cells exposure under similar tested conditions**.** The induction of staphylocidal activity by OBN was attained at lower aminoglycosides concentrations by 4–16 folds compared to their individual doses. Consequently, this lower dose requirement would be further helpful to decrease the clinical side effects of aminoglycoside as it causes ototoxicity affecting the vestibulo-cochlear system or nephrotoxicity. Clinically, aminoglycoside therapy for the treatment of staphylococcal infections in the inner wall endocarditis causes nephrotoxicity [43]. Other clinical limitation of combined therapy of aminoglycosides causes azotemia by increasing the level of nitrogenous substances such as urea, creatinine, various body waste compounds in the blood [44]. Investigations of more than one therapeutic agent in combination have been explored by the different set of studies [16, 17] and pragmatically a lucrative approach for the treatment of life-threatening resistant strains of *S. aureus.* The augmentation in drug potency of aminoglycosides has been previously reported to search an alternative treatment strategy against drug sensitive and resistant *S. aureus*.

Many studies have highlighted that cell membrane-targeted agent or membrane potential factors enhanced the aminoglycoside uptake [16, 45, 46]. In order to investigate the aminoglycoside uptake, the intracellular GEN concentration was measured in presence of OBN. OBN that substantially induces the cell killing with aminoglycosides (GEN, KAN, and AMK), also potentiate GEN uptake by 66% as compared to control (without OBN). The enhancement of GEN uptake was further corroborated by reenergizing the starved cells with glucose (10 mM) which remarkably increased GEN uptake by 50% with OBN. In contrast, the degree of GEN uptake was hampered and found to be similar to control when OBN treated cells were subsequently exposed with the crude membrane, isolated from *S. aureus*. Thereby, the GEN uptake was associated with the disturbance of cell membrane integrity and membrane integrity was somewhat mended upon addition of exogenous crude membrane. Likewise, OBN with GEN led to leakage of bacterial ATP showed that the membrane damaged which was also confirmed by nitrocefin assay. The consequence of *S. aureus* killing was associated with the accumulation of intracellular GEN upon alteration of membrane permeability by disturbing cell integrity [16].

Encasement of biofilm over cell boundary causes a major hurdle in the effective treatment as antibiotics failure to enter in the cells, resulting to emerge resistance to antibiotics and immune defence. Despite to higher drug-resistance, biofilm-forming cells tend to adhere to implanted medical devices and human tissues that majorly contribute to the acute and chronic infections. With a note, the biofilm attachment was disrupted by almost 80 and 50% with 5 mg/mL and 1.5 mg/mL OBN, respectively. The biofilm detachment was further validated by FITC and or blood plasma coated *S. aureus* cells analysis. OBN also showed inhibition in biofilm formation and it was inhibited by 50 and 90% at 5 mg/mL (MBIC_50_) and 10 mg/mL (MBIC_90_) OBN. Of note, the inhibition of biofilm formation tends to increase upon longer exposure over 48 h OBN treatment. The biofilm inhibitory effect has reported previously in different studies [31]. Formation of biofilm is stable at near neutral pH and steadily decreases under acidic pH. Formation of biofilm investigated with OBN in the presence or absence of GEN at 7.0, 6.0, 5.5 pH. As expected, the formation of biofilm was substantially reduced by 21 ± 4%, 37 ± 5% and 55 ± 5% at 7.0, 6.0 5.5 pH respectively upon OBN (2 mg/mL) treatment. At pH 7.0, biofilm inhibition was enhanced by 67 ± 5% and no such effect was observed at pH 6.0 and 5.5 when both OBN added with GEN. Therefore, *S. aureus* showed resistant to GEN at lower pH. Earlier study revealed that resistance of *S. aureus* to GEN is related to pH which affects the proton motive force. The uptake of GEN decreased at low pH is due to a decrease in the ATP level inside the cell [47].

## Conclusions

Taken together, we established that OBN synergizes the antimicrobial activity of aminoglycosides that induces cell killing due to intracellular accumulation of GEN by disturbing cell homeostasis. It may be proven an effective approach for the treatment of staphylococcal infections.

## Data Availability

Data sharing not applicable to this article as no datasets were generated or analysed during the current study.

## Abbreviations

AMK: Amikacin
AMP: Ampicillin
ATP: Adenosine 5′-triphosphate
FIC: Fractional inhibitory concentration
FICI: Fractional inhibitory concentration index
FITC: Fluorescein iso-thiocyanate
GEN: Gentamycin
KAN: Kanamycin
MBIC: Minimal bioflim inhibitory concentration
MDR: Multi-drug resistance
MIC: Minimal inhibitory concentration
MRSA: Methicillin-resistant *S. aureus*
MSSA: Methicillin-sensitive *S. aureus*
OBN: Ouabain
TET: Tetracycline
VAN: Vancomycin
